# Primary weight loss failure after Roux-en-Y gastric bypass is characterized by impaired gut-hormone mediated regulation of food intake

**DOI:** 10.1038/s41366-023-01372-8

**Published:** 2023-08-31

**Authors:** Kirstine Nyvold Bojsen-Møller, Maria Saur Svane, Christoffer Martinussen, Carsten Dirksen, Nils Bruun Jørgensen, Jens-Erik Beck Jensen, Christian Zinck Jensen, Signe Sørensen Torekov, Viggo Bjerregaard Kristiansen, Jens Frederik Rehfeld, Jette Bork-Jensen, Niels Grarup, Torben Hansen, Bolette Hartmann, Jens Juul Holst, Sten Madsbad

**Affiliations:** 1 Dept. of Endocrinology, Copenhagen University Hospital Hvidovre, Copenhagen, Denmark; 2 Novo Nordisk Foundation Center for Basic Metabolic Research, University of Copenhagen, Copenhagen, Denmark; 3 Dept. of Clinical Medicine, University of Copenhagen, Copenhagen, Denmark; 4 Dept. of Biomedical Sciences, University of Copenhagen, Copenhagen, Denmark; 5 Dept. of Surgical Gastroenterology, Copenhagen University Hospital Hvidovre, Copenhagen, Denmark; 6 Dept. of Clinical Biochemistry, Rigshospitalet, Copenhagen, Denmark

**Keywords:** Obesity, Weight management, Genetics, Obesity

## Abstract

**Background/Objectives:**

After Roux-en-Y gastric bypass (RYGB) a subset of patients never obtain excess BMI loss (EBMIL) > 50% and are categorized as having primary weight loss (WL) failure. We hypothesized that postprandial concentrations of glucagon-like peptide 1 (GLP-1) and peptide YY (PYY) would be lower in patients with primary WL failure compared with patients with successfully maintained WL. Furthermore, that inhibition of gut hormone secretions would increase ad libitum food intake less in patients with primary WL failure.

**Subjects/Methods:**

Twenty women with primary WL failure (LowEBMIL < 50%) were individually matched to twenty women with successful WL (HighEBMIL > 60%) on age, preoperative BMI and time from RYGB. On separate days performed in a random order, patient-blinded subcutaneous injections of octreotide or saline (placebo) were followed by a fixed breakfast and an ad libitum lunch with blood sampling for appetite regulating hormones and Visual-Analogue-Scale (VAS)-scoring of hunger/satiety. Furthermore, participants underwent gene variant analysis for GLP-1, PYY and their receptors, indirect calorimetry, dual-energy X-ray absorptiometry (DXA)-scans, 4-days at-home food registration and 14-days step counting.

**Results:**

On placebo days, postprandial GLP-1, PYY and cholecystokinin (CCK) concentrations were similar between groups after breakfast. Fasting ghrelin was lower in LowEBMIL, but the postprandial suppression was similar. LowEBMIL had lower satiety VAS-scores and less suppression of hunger VAS-scores. Gene variants did not differ between groups. Octreotide diminished GLP-1, PYY, CCK and ghrelin concentrations in both groups. Octreotide did not affect ad libitum food intake in LowEBMIL (−1% [−13, 12], mean [95%CI]), while food intake increased in HighEBMIL (+23% [2,44]).

**Conclusions:**

Primary WL failure after RYGB was not characterized by impaired secretions of appetite regulating gut hormones. Interestingly, inhibition of gut hormone secretions with octreotide only increased food intake in patients with successful WL post-RYGB. Thus, an impaired central anorectic response to gut hormones may contribute to primary WL failure after RYGB.

## Introduction

Roux-en-Y gastric bypass (RYGB) induces a large and long-term maintained weight loss (WL) in most patients; 75% of patients obtain and maintain >50% excess BMI loss (EBMIL) for at least 5 years [1, 2]. In the remaining group with suboptimal long-term WL, the majority experience weight regain after an initial successful WL (20% of all operated), whereas only 5% of all operated patients never obtain >50% EBMIL and are classified as having primary WL failure [1].

Preoperative predictors of a suboptimal WL after RYGB include type 2 diabetes [1–6], higher initial BMI [1–3, 5] and higher age [2, 4, 6], and post-bariatric WL may also be influenced by genetic factors [5, 7, 8], whereas low socioeconomic status does not seem to predict less WL [9]. Nevertheless, these factors only explain a minor proportion of the WL response [2, 6, 8], and specific risk factors for primary WL failure after RYGB have been sparsely investigated. RYGB is characterized by marked alterations in the secretory profile of gut hormones known to influence appetite with consistent findings of higher postprandial concentrations of the anorexigenic glucagon-like peptide 1 (GLP-1), peptide YY (PYY), cholecystokinin (CCK) and lower concentrations of the orexigenic ghrelin after surgery [10–13]. A causal link between the higher anorexigenic hormone concentrations and post-RYGB appetite control has been supported by studies, where ad libitum food intake is increased after inhibition of hormone secretions by the somatostatin analogue octreotide in humans [10, 14] and rats [15] or after combined inhibition of GLP-1 and PYY actions in humans [16]. Also, a study applying functional magnetic resonance imaging (fMRI) in combination with octreotide supports a specific role of the anorexigenic gut hormones for brain reward responses to food after RYGB [17]. We and others have previously investigated the role of gut hormones for WL responses after RYGB, and lower postprandial GLP-1 and less suppression of ghrelin have been reported in patients with suboptimal WL compared with patients with successful WL in most [10, 14, 18, 19] but not all studies [20]. However, these studies did not distinguish between patients with primary WL failure or weight regain [10, 18–20] or included only patients with weight regain [14].

Therefore, we aimed at investigating the role of gut hormones for appetite regulation in patients with primary WL failure after RYGB. We hypothesized that patients with primary WL failure would have lower postprandial concentrations of GLP-1 and PYY (primary endpoint) compared with patients, who had maintained a successful WL and were carefully matched for age, sex, preoperative BMI and time from surgery. Secondly, we hypothesized that inhibition of gut hormone secretions with octreotide would increase ad libitum food intake *less* in patients with primary WL failure compared with patients with successful WL after RYGB (secondary endpoint). In addition, variants in the genes for GLP-1, PYY and their receptors were analyzed.

## Subjects and Methods

### Subjects

Eligible patients were identified using a database of > 600 patients operated with RYGB at Copenhagen University Hospital Hvidovre, Denmark in 2012–2015. Patients with primary WL failure (LowEBMIL, *n* = 20) were defined by postoperative EBMIL < 50% at all visits including 1 and 2 years postoperatively after uncomplicated RYGB. Each patient in the primary WL failure group was matched individually with one patient with successful WL (postoperative EBMIL > 60%, HighEBMIL, *n* = 20) with respect to age, preoperative BMI and time from RYGB. Only women without a history of diabetes (Hba1c < 48 mmol/mol without glucose-lowering medication) were included. Exclusion criteria were unstable weight (self-reported > ±3 kg in 3 months), inadequately treated hypothyroidism, use of antithyroid medication or medication affecting appetite, hemoglobin < 6.5 mmol/L, pregnancy/breastfeeding or unwillingness/allergies toward the test meals. The study was approved by the Regional Ethical Committee of the Capital Region (H-4–2014–007), by the Danish Data Protection Agency and was performed in accordance with the Helsinki declaration and registered at ClinicalTrials.gov (NCT02344632). Written informed consent was obtained from all participants before inclusion.

### Methods

Participants underwent three experimental test days separated by ≥ 3 days at Copenhagen University Hospital Hvidovre: Two meal tests with patient-blinded subcutaneous injection of octreotide or placebo in randomized order and on a third day whole-body dual-energy X-ray absorptiometry (DXA) scan (Discovery A, S/N 83487; Hologic Inc., Bedford, MA using the Apex 5.6.05 software) after an overnight fast. At home, four days (3 weekdays, 1 weekend-day) of complete food registration and two weeks of patient-blinded step counting (Omron walking style pro 2.0, Bannockburn, IL, USA) were performed. Calorie content and macronutrient composition was registered by a dietician after interviewing the participant.

### Meal test days

Participants refrained from strenuous physical activity and alcohol for 3 days prior to test days and ingested identical diets on the day before. After an overnight fast (10–12 h), participants were weighed and seated reclined in a hospital bed allowing no strenuous activity. An antecubital vein catheter was inserted for blood sampling. After sampling of three fasting blood samples, octreotide (Octreotide Hospira, Hospira Nordic, Denmark [12 LowEBMIL, 13 HighEBMIL] or Sandostatin, Novartis, Denmark [8 LowEBMIL, 7 HighEBMIL]) 1 μg/kg bodyweight (max 100 μg) or a similar volume of saline (placebo) was injected subcutaneously at T = −30 min. Two basal blood samples were drawn before (T = −10 and 0 min) serving the breakfast (at T = 0), which consisted of ½ slice of whole meal toast with 1 slice of cheese, margarine spread and marmalade, 2 dl yoghurt with 20 g oatmeal, 16 raisins, and 5 almonds (Energy content: 1523 kJ, 53E% carbohydrate, 33E% fat, and 14E% protein) and was ingested evenly over 20 minutes finishing with 100 ml of water. To estimate intestinal nutrient entry, 1 g of paracetamol (Pamol; Nycomed, Roskilde, Denmark) was added to the meal portion ingested within the first 5 min. At T = 240 min, the ad libitum lunch of thoroughly mixed pasta Bolognese (Energy content 533 kJ/100 g, 53E% carbohydrate, 14E% protein and 33E% fat) was served. Patients were instructed to eat until pleasantly satiated. Water (100 mL) was allowed with the meal. The meal was weighed before and after serving to estimate ad libitum food intake. Blood was sampled at fixed intervals until T = 300 min along with assessment of blood pressure (BP) and pulse rates. Visual Analogue Scale (VAS)-scoring for hunger and satiety was performed at T = − 30 and 0 min and with 30–60 min intervals by marking on a line of 100 mm with a text expressing the most positive and the most negative rating anchored at each end. Participants could not compare with previous ratings or discuss ratings with others. Indirect calorimetry (20 min) using a canopy system (Deltatrac II Metabolic Monitor, Datex-Ohmeda, Helsinki, Finland) was performed twice at T = − 30 and 30 min.

### Sample collection and laboratory analyses

Blood was collected into chilled EDTA tubes to which were added a DPP4 inhibitor (valine-pyrrolidide, final concentration of 0.01 mM; gift from Novo Nordisk, Bagsværd, Denmark) and aprotinin (final concentration 0.01 mmol/l). Plasma was stored for batch analysis of total GLP-1 using antiserum 89390 and glucagon using the C-terminal antibody code 4305 (both RIA) and total PYY using ELISA (EZHPYYT66K, Millipore, USA). CCK was measured in EDTA-plasma with RIA [21]. Active ghrelin was measures in EDTA-plasma treated with 1 N hydrochloric acid (5 µL/ml plasma) and phenylmethylsulfonylfluoride (20 µg/ml plasma) using RIA (GHRT-88HK, Millipore, Billerica, MA, USA). Serum C-peptide concentrations were determined by Immulite 2000 analyzer (Siemens Healthcare Diagnostics Inc., Tarrytown, NY, USA). Concentrations of paracetamol, leptin and the soluble leptin receptor were analyzed in EDTA-plasma using Cobas immunoassay (Roche Diagnostics, Rotkreuz, Switzerland), RIA (HL-81K, EMD Millipore, St. Louis, Missouri, USA) and ELISA (Human Leptin R Immunoassay, Quantikine, Minneapolis, Minnesota, USA), respectively. Buffy coat was stored for later chip genotyping using the Global Screening Array-24 (v2.0) (Illumina, San Diego, CA, USA).

### Calculations

Fasting and basal concentrations were calculated as the mean of samples taken prior to injection of octreotide/placebo and prior to ingestion of the breakfast meal, respectively. The area-under-the-curves (AUCs) were calculated using the trapezoidal rule for the fixed breakfast (T = 0–240 min) and the ad libitum meal (T = 240–300 min) separately. HOMA2-IR was estimated from fasting glucose and C-peptide concentrations using spreadsheets available at www.dtu.ox.ac.uk/homacalculator. Basal energy expenditure (BEE) was calculated from the median oxygen uptake (V̇O2) and carbon dioxide output (V̇CO2) in the basal period on the placebo day: BEE = ([3.9·V̇O2] + [1.1·V̇CO2])·1.44 [22]. Respiratory exchange ratios (RER = V̇CO2/V̇O2) were calculated for the basal and postprandial period for both test days.

### Statistics

The primary outcome was comparison of gut hormone responses (AUC of GLP-1 and PYY) between groups after breakfast on the placebo day, whereas the effect of octreotide on ad libitum food intake was a prespecified secondary outcome. Data were analyzed by ANOVA in a linear mixed effects model using group (LowEBMIL vs HighEBMIL), octreotide (placebo vs octreotide) and the interaction between group x octreotide as fixed effects and individual subjects as random effect. A match variable specifying the pairing of the subjects was included as fixed effect. Analysis of AUC was performed with and without the pre-meal concentration (basal concentration or the concentration at T = 240 for AUC breakfast and AUC ad libitum, respectively) as fixed effect reporting readouts from both models. Variables were logarithmically transformed if required to optimize model fit. P-values for the following comparisons were reported: LowEBMIL vs HighEBMIL on placebo days, main effect of octreotide and the group x octreotide interaction. In case of significant interaction, the following post hoc comparisons were added: LowEBMIL vs HighEBMIL on octreotide days and the octreotide response within groups. Participants’ characteristics were analyzed by ANOVA using group and the match variable as fixed effects. Statistical analysis was performed in R v.3.5.2 (www.R-project.org) using the “nlme”-package. A p-value < 0.05 was considered significant.

### Gene analyses

Genotypes were called using Illuminas GenCall algorithm and subjected to standard quality control (QC). All samples were good quality (no mislabeled sex, no outlying heterozygosity, sample call rate > 98%). Variants with call rate < 98%, out of Hardy-Weinberg equilibrium (*p* > 10^−5^) and monomorphic variants were excluded. Imputation was done using the Michigan imputation server pipeline 1.5.7 (www.imputationserver.sph.umich.edu/index) with Eagle v2.4 for phasing and Minimac4 for imputation with the HRC1.1 panel. Individual polygenic scores (PGS) were calculated using a weighted score for BMI of 2.1 M Single Nucleotide Polymorphisms (SNPs) [23] and including 99% of the variants. Common variants (minor allele frequency> 5%) of high quality (R2 < 0.8) in the glucagon (GCG), PYY, GLP-1 Receptor (GLP1R) and Neuropeptide Y2 Receptor (NPY2R) gene loci were extracted (from transcription start site −500 kb to end site +250 kb) and annotated using VEP [24]. For each variant the effect of being a carrier on the probability of being in the HighEBMIL versus the LowEBMIL group was tested using conditional logistic regression models in R v.4.0.2 using clogit from “survival”-package v.3.1–12. The significance threshold was adjusted with the effective number of markers (*M*_eff_) [25] using the “poolr”-package resulting in a threshold of p_adj_ = 0.0009.

## Results

### Participants’ characteristics (Table 1)

**Fig. 1:**
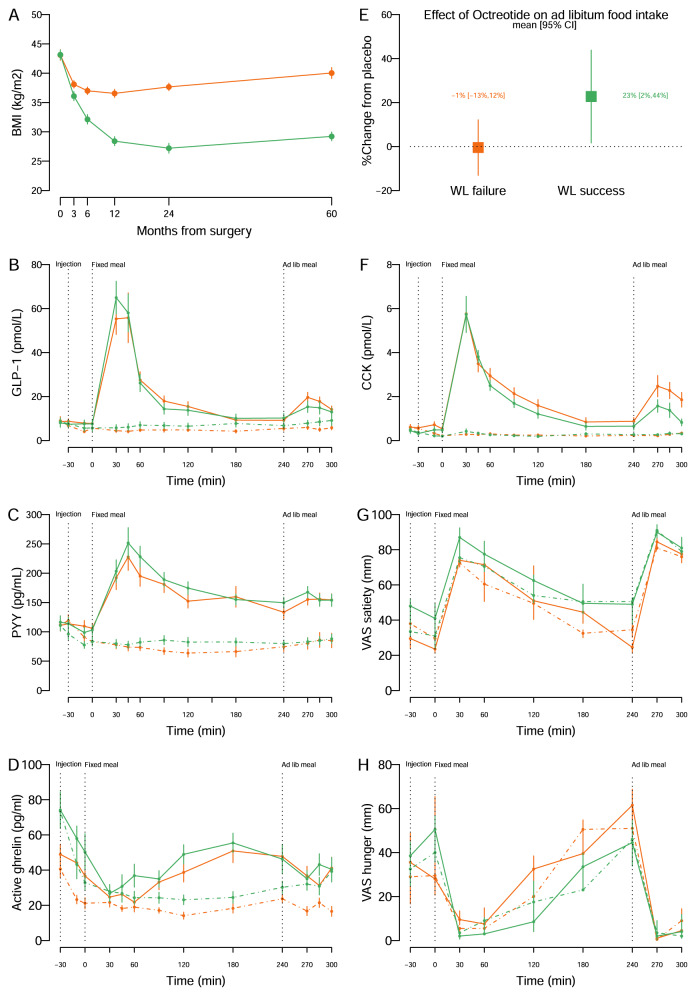
Weight loss and measures of appetite regulation in patients with primary weight loss failure (LowEBMIL group, orange) and patients with successful weight loss (HighEBMIL group, green) after Roux-en-Y gastric bypass. **A** Postoperative body mass index (BMI) (mean ± sem). **B** Plasma total GLP-1 (mean ± sem). **C** Plasma total PYY (mean ± sem). **D** Plasma active ghrelin (mean ± sem). **E** Relative effect (%) of octreotide versus placebo on ad libitum food intake at lunch (mean [95CI]). **F** Plasma CCK (mean ± sem). **G** Visual analogue scale (VAS) scores of satiety (median, 40–60 percentiles). **H** Visual analogue scale (VAS) scores of hunger (median, 40–60 percentiles). In panels (**B**–**D**) and (**F**–**H**), solid lines represent responses on the placebo day, whereas dashed lines represent responses on the octreotide day after breakfast (ingested at *t* = 0 min) and ad libitum lunch meal (ingested at *t* = 240 min).

Participants were women aged 51 ± 9 (mean, SD) years with a preoperative BMI of 43 ± 4 kg/m^2^. They were examined at a median of 5 years post-RYGB surgery, at which time the LowEBMIL group had an EBMIL of 23% compared with 74% in the HighEBMIL group, equivalent to a 10 BMI-points difference between groups (Fig. 1A).

#### Metabolic control, body composition and energy expenditure

Hba1c and HOMA2-IR were slightly higher in the LowEBMIL who had approximately 20 kg more fat and 10 kg more lean mass compared with the HighEBMIL group. Fasting leptin concentration was higher in LowEBMIL, but the leptin concentration expressed per kg fat mass did not differ between groups (*p* = 0.327). Circulating concentrations of the leptin receptor did not differ between groups. Basal energy expenditure (BEE) was higher in LowEBMIL, but without difference between groups (*p* = 0.226) when adjusting for lean mass in the ANOVA.

#### Self-reported food intake and activity

At home food intake during free-living did not differ between groups, neither with respect to energy content nor macronutrient composition. Daily median steps were low and without significant differences between groups.

### Gut hormones, appetite evaluation and gene variants

#### Total GLP-1 (Fig. 1B)

Fasting GLP-1 was similar between groups. On placebo days, no differences were observed between groups in the GLP-1 response (AUC and peak) after breakfast (Table 2) or after ad libitum lunch (Supplementary Table 1). Octreotide effectively inhibited GLP-1 secretion after breakfast and after ad libitum lunch in both groups. The effect of octreotide on GLP-1 after breakfast was similar between groups (Table 2), whereas GLP-1 after the ad libitum meal tended to be higher in the HighEBMIL group after octreotide for both AUC and peak (9 pmol/L [6;13] vs 6 [4;10], median [IQR], *p* = 0.051) (Supplementary Table 1). The GLP-1 concentration at initiation of the ad libitum meal (*t* = 240) was similar between groups on the placebo as well as the octreotide day (*p* = 0.180).

#### Total PYY (Fig. 1C)

Fasting and postprandial PYY concentrations (AUC and peak) were similar between groups on placebo days after both the breakfast and ad libitum meal including at *t* = 240 (Table 2 and Supplementary Table 1, respectively). Octreotide lowered the PYY response similarly in both groups in response to breakfast and ad libitum lunch.

#### Active ghrelin (Fig. 1D)

Fasting ghrelin was lower in LowEBMIL. After breakfast on the placebo day, ghrelin was suppressed in both groups resulting in similar AUC (with and without correction for basal concentrations) and nadir concentrations (Table 2). Pre-meal, AUC and nadir of ghrelin concentrations in relation to the ad libitum meal were also similar between groups on the placebo day (Supplementary Table 1). Octreotide decreased ghrelin similarly between groups after breakfast (Table 2). In relation to the ad libitum meal, octreotide lowered the pre-meal ghrelin concentration similarly in both groups, whereas AUC tended to be lowered more by octreotide in LowEBMIL (*p* = 0.079) resulting in lower nadir concentrations (10 pg/mL [4;16] vs. 19 [12;30], median [IQR], *p* = 0.026) (Supplementary Table 1).

#### CCK (Fig. 1F)

Fasting CCK tended to be higher in LowEBMIL, but neither AUC nor peak differed between groups after breakfast on placebo days (Table 2). In relation to the ad libitum meal, pre-meal CCK was similar but AUC and peak concentrations were higher in LowEBMIL on placebo days (Supplementary Table 1). Octreotide diminished CCK in response to breakfast similarly between groups (Table 2) and suppressed pre-meal, AUC and peak CCK in response to the ad libitum meal to a similar level between groups (Supplementary Table 1).

#### Ad libitum food intake (Fig. 1E)

Ad libitum meal intake did not differ between groups on placebo days (Table 2). The response to octreotide differed significantly between groups; in the HighEBMIL group ad libitum meal intake increased after ocetrotide by 23%, whereas octreotide had no effect on food intake in the LowEBMIL group (Fig. 1E). Ingestion time neither differed between groups on placebo days (LowEBMIL: 10 min [9;12], HighEBMIL: 9 min [7;12], median [IQR]) nor after octreotide (LowEBMIL: 10 [9;11], HighEBMIL: 9 [7;10]).

#### VAS-scores (Fig. 1G, H)

On placebo days, satiety and hunger scores were similar between groups in the fasting/basal state but satiety scores were lower and hunger scores less suppressed after breakfast in LowEBMIL (Table 2) resulting in lower satiety scores before the ad libitum meal in LowEBMIL (25 mm [18;49] vs 49 [23;70], median [IQR], *p* = 0.021) (Supplementary Table 1). VAS-scores after the ad libitum meal did not differ between groups when correcting for differences in pre-meal levels (Supplementary Table 1). Octreotide tended to lower satiety scores equally in both groups after breakfast, whereas hunger scores were not affected.

#### Gene variants

Polygenic scores for BMI did not differ between groups (Table 1). 5640 gene variants were identified in the loci for genes encoding GLP-1 and PYY and their receptors (Supplementary Table 2), but none of the variants reached the adjusted significance threshold (lowest *p*-value *p* = 0.03, Supplementary Fig. 1A–D for regional plots).

**Table 1:** Participants’ characteristics.

	Primary WL failure (LowEBMIL)	Successful WL (HighEBMIL)	*P*-value
*N*	20 women	20 women	na
Age at study (years)	50 [47;57]	50 [44;58]	na
Time from surgery (years)	5 [3;6]	5 [4;6]	na
Preoperative BMI (kg/m^2^)	42.4 [40.2;45.8]	42.2 [40.8;45.4]	na
Max postoperative EBMIL recorded (%)	34% [27;41]	80% [76;85]	<0.001
BMI at study (kg/m^2^)	39.4 [37.5;42.0]	29.4 [28.2;31.1]	<0.001
EBMIL at study (%)	23% [9;27]	74% [70;83]	<0.001
Body weight at study (kg)	109.9 [99.7;115.4]	78.9 [74.7;84.3]	<0.001
Weight loss at study (kg, % of total body weight)	9.5 kg [4.5;13], 8.6% [3.8;11]	36 kg [32;44], 32% [28;36]	<0.001, <0.001
Fat mass at study (kg, % of body weight)	48 kg [44;55], 46% [44;49]	29 kg [28;32], 38% [36;41]	<0.001, <0.001
Lean mass at study (kg)	55 [52;60]	44 [42;49]	<0.001
Polygenic score for BMI	38.86 [38.79;38.94]	38.92 [38.81;39.04]	0.358
Hba1c at study (mmol/mol)	37 [36;40]	35 [33;37]	0.003
HOMA2-IR at study fasting	1.5 [1.2;2.2]	1.0 [0.8;1.2]	<0.001
P-leptin (ng/mL) fasting	79 [56;106]	34 [28;40]	<0.001
P-leptin receptor (ng/mL) fasting	23 [19;28]	25 [21;34]	0.436
Basal energy expenditure (kcal/day) resting	1551 [1481;1672]	1414 [1284;1478]	0.006
Steps per day	3204 [2800;6540]	4619 [3607;5763]	0.437
Self-reported at home energy intake (kcal/day)	1705 [1307;2038]	1655 [1311;2151]	0.696
Self reported energy % from carbohydrates	45 [42;47]	43 [37;49]	0.869
Self reported energy % from protein	19 [16;22]	20 [16;24]	0.887
Self reported energy % from fat	36 [31;38]	36 [31;42]	0.981

Median [IQR], *na* Not assessed (matching variables).

*EBMIL* Excess BMI loss = BMIpreoperative-BMIpostoperative/BMIpreoperative-25, *HOMA2-IR* Homeostasis Model Assessment 2 of insulin resistance (C-peptide based).

**Fig. 2:**
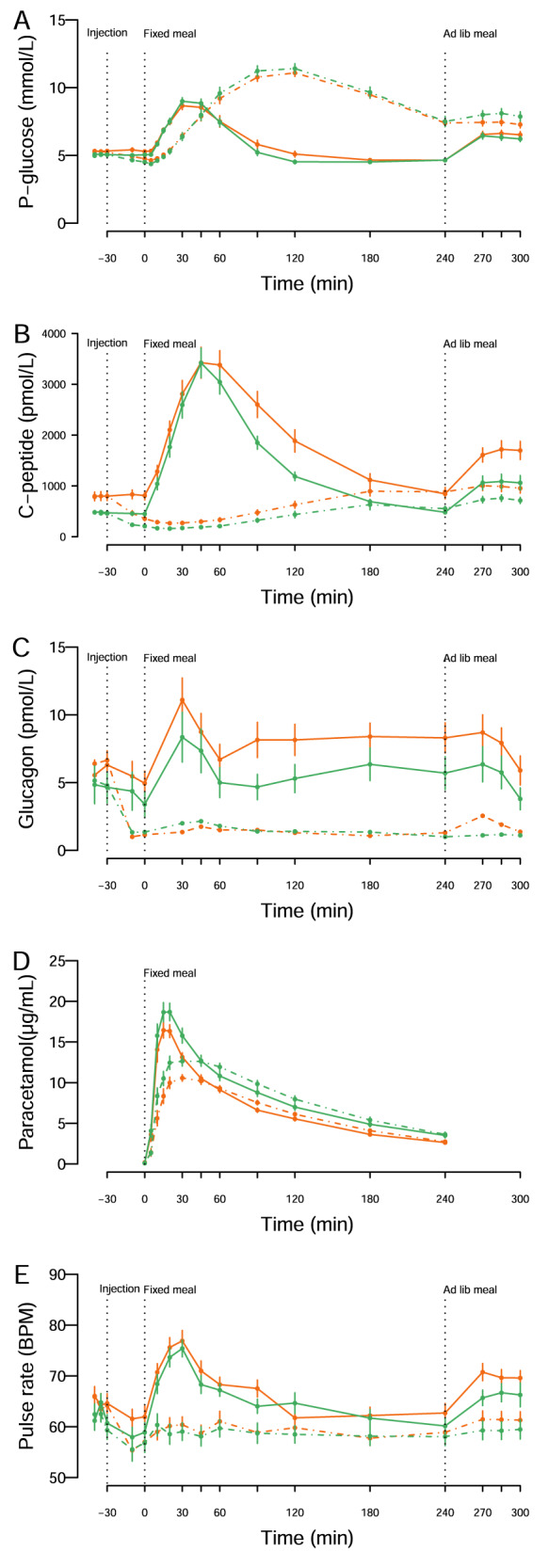
Metabolic parameters, gastric emptying, and pulse rates in patients with primary weight loss failure (LowEBMIL group, orange) and patients with successful weight loss (HighEBMIL group, green) after Roux-en-Y gastric bypass. **A** Plasma glucose (mean ± sem). **B** Serum C-peptide (mean ± sem). **C** Plasma glucagon (mean ± sem). **D** Plasma paracetamol (mean ± sem). **E** Pulse rates (BPM, beats per minutes) (mean ± sem). Solid lines represent responses on the placebo day, whereas dashed lines represent responses on the octreotide day after breakfast (ingested at *t* = 0 min) and ad libitum lunch meal (ingested at *t* = 240 min).

### Metabolic parameters

#### Glucose and C-peptide (Fig. 2A, B)

Fasting and basal glucose concentrations were slightly higher in LowEBMIL, but postprandial glucose concentrations were similar between groups after breakfast (Table 2) and ad libitum lunch (Supplementary Table 1) on placebo days. Octreotide lowered basal glucose and increased postprandial glucose concentrations similarly in both groups resulting in similar pre-meal plasma glucose between groups at the time of the ad libitum serving.

**Table 2:** Appetite regulating hormones and measures of glucose metabolism in response to the breakfast meal.

	Primary WL failure (LowEBMIL) Placebo	Successful WL (HighEBMIL) Placebo	*P*-value	Primary WL failure (LowEBMIL) Octreotide	Successful WL (HighEBMIL) Octreotide	Main Oct	Group x Oct
GLP-1 fasting (pmol/L)	8 [4;14]	5 [4;9]	0.299	7 [4;10]	6 [3;12]	0.832	0.519
GLP-1 basal (pmol/L)	7[5;10]	6 [3;9]	0.365	4[2;7]	3 [1;6]	0.001	0.799
GLP-1 AUC_0–240_ (pmol·L^−1^·min)	4039 [3336;6018]	4669 [3006;5891]	0.876 0.983^a^	908 [476;1708]	1136 [789;1708]	<0.001 <0.001^a^	0.140 0.128^a^
GLP-1 peak (pmol/L)	52 [40;66]	64 [37;93]	0.460	8 [6;12]	9 [6;13]	<0.001	0.852
PYY fasting (pg/mL)	104 [86; 130]	110 [77;143]	0.884	105 [83;135]	96 [72;127]	0.316	0.267
PYY basal (pg/mL)	95 [79;126]	97 [79;120]	0.870	84 [66;111]	80 [58;108]	<0.001	0.825
PYY AUC_0–240_ (ng·mL^−1^·min)	34 [31;48]	40 [34;47]	0.337 0.193^a^	15 [12;21]	18 [14;25]	<0.001 <0.001^a^	0.440 0.402^a^
PYY peak (pg/mL)	238 [172;322]	233 [184;297]	0.392	85 [72;109]	88 [64;137)	<0.001	0.869
CCK fasting (pmol/L)	0.5 [0.4;0.7]	0.3 [0.2;0.6]	0.083	0.5 [0.3;0.7]	0.5 [0.3;0.5]	0.381	0.363
CCK basal (pmol/L)	0.5 [0.2;0.8]	0.2 [0.2;0.4]	0.067	0.2 [0.1;0.4]	0.1 [0.1;0.3]	<0.001	0.509
CCK AUC_0–240_ (pmol·L^−1^·min)	423 [347;620]	371 [283;462]	0.433 0.779^a^	53 [32;79]	46 [32;86]	<0.001 <0.001^a^	0.494 0.597^a^
CCK peak (pmol/L)	5.5 [4.0;7.2]	4.7 [3.9;6.2]	0.913	0.5 [0.3;0.7]	0.5 [0.3;0.8]	<0.001	0.464
Ghrelin fasting (pg/mL)	48 [35;57]	62 [39;103]	0.022	32 [28;50]	57 [49; 112]	0.188	0.265
Ghrelin basal (pg/mL)	36 [29;51]	50 [27;75]	0.051	21 [16;31]	30 [21;50]	0.001	0.876
Ghrelin AUC_0–240_ (ng ·mL^−1^·min)	8.8 [6.4;12.4]	10.2 [8.2;12.5]	0.138 0.691^a^	4.8[2.9;5.9]	5.5[4.5; 7.9]	<0.001 <0.001^a^	0.737 0.602^a^
Ghrelin nadir (pg/mL)	14 [9;21]	15 [6;24]	0.330	8 [4;12]	14 [4;21]	0.001	0.683
VAS_satiety_ fasting (mm)	30 [13;47]	48 [31;65]	0.161	38 [23;54]	34 [25;47]	0.977	0.178
VAS_satiety_ basal (mm)	24 [14;54]	41 [23;63]	0.427	30 [18;49]	31 [20;52]	0.385	0.778
VAS_satiety_ AUC_0–240_ (cm ·min)	1228 [966;1664]	1406 [1278;2023]	0.049 0.036^a^	1072 [905;1510]	1283 [1109;1896]	0.064 0.052^a^	0.948 0.917^a^
VAS_hunger_ fasting (mm)	36 [10;54]	39 [16;52]	0.730	29 [13;58]	33 [14;47]	0.890	0.783
VAS_hunger_ basal (mm)	28 [15;74]	51 [18;63]	0.896	30 [18;69]	40 [27;66]	0.718	0.718
VAS_hunger_ AUC_0–240_ (cm ·min)	835 [345;1172]	581 [134;1001]	0.224 0.032^a^	751 [279;1073]	713 [296;924]	0.879 0.843^a^	0.428 0.484^a^
Glucose fasting (mmol/L)	5.2 [5.0;5.5]	5.1 [4.9;5.2]	0.063	5.2 [5.0;5.3]	5.0 [4.9;5.2]	0.248	0.984
Glucose basal (mmol/L)	5.3 [5.1;5.5]	5.1 [4.9;5.1]	0.009	4.8 [4.5;5.0]	4.6 [4.4;4.8]	<0.001	0.540
Glucose AUC_0–240_ (mmol·L^−1^·min)	1368 [1283;1558]	1357 [1290;1407]	0.537 0.756^a^	2137 [1967;2217]	2182 [1976;2395]	<0.001 <0.001^a^	0.266 0.336^a^
Glucose peak (mmol/L)	9.1 [8.7;9.6]	9.5 [8.5;10.5]	0.519	11.3 [10.5;11.7]	11.4 [10.6;12.7]	<0.001	0.918
C-peptide fasting (pmol/L)	677 [557;969]	445 [414;519]	< 0.001	679 [550;927]	474 [373;518]	0.336	0.520
C-peptide basal (pmol/L)	733 [507;1000]	417 [376;484]	< 0.001	320 [247;493]	227 [187;257]	<0.001	0.323
C-peptide AUC_0–240_ (nmol·L^−1^·min)	411 [356;542]	337 [274;393]	0.039 0.760^a^	130** [88;186]	88**^##^ [73;99]	<0.001 <0.001^a^	0.133 0.021^a^
C-peptide peak (pmol/L)	3211 [2411;4618]	3509 [2718;3981]	0.835	897** [570;1260]	588**^#^ [454;692]	<0.001	0.027
Glucagon fasting (pmol/L)	4.3 [2.3;9.5]	2.8 [1.0;6.0]	0.211	5.5 [2.8;9.6]	2.8 [1.9;6.5]	0.272	0.752
Glucagon basal (pmol/L)	3.8 [1.0;8.9]	1.8 [1.0;3.9]	0.107	1.0 [1.0;1.0]	1.0 [1.0;1.0]	<0.001	0.176
Glucagon AUC_0–240_ (pmol·L^−1^·min)	1823 [1273;2666]	1309 [471;1761]	0.019 0.031^a^	240** [240;294]	240** [240;315]	<0.001 <0.001^a^	0.033 0.057^a^
Glucagon peak (pmol/L)	15 [10;18]	9 [4;12]	0.071	1.0** [1.0;2.2]	1.0**[1.0;3.0]	<0.001	0.044
PCM Time to peak (min)	15 [10;20]	15 [15;20]	0.516	30 [20;38]	30 [20;45]	<0.001	0.770
Pulse rate fasting (bpm)	65 [59;71]	63 [53;67]	0.133	66 [61;70]	63 [56;69]	0.773	0.947
Pulse rate basal (bpm)	61 [55;68]	60 [52;64]	0.261	56 [52;61]	57 [50;61]	<0.001	0.214
Pulse rate peak (bpm)	77 [72; 84]	77 [73;80]	0.901	67 [58;71]	65 [60;68]	<0.001	0.788
RER basal	0.80 [0.78;0.84]	0.78 [0.74;0.81]	0.068	0.74 [0.70;0.80]	0.71 [0.69;0.80]	<0.001	0.528
RER early meal	0.89 [0.86;0.93]	0.87 [0.84;0.90]	0.292	0.77 [0.72;0.80]	0.76 [0.72;0.79]	<0.001	0.245
Ad libitum lunch meal intake (kJ)	1391 [1141;1826]	1279 [815;1450]	0.183	1277 [995; 1515]	1354* [1018;1722]	0.279	0.046

Median [IQR]. ^a^Model adjusted for pre-meal concentrations/levels, *p < 0.050, **p < 0.010 compared with placebo day, ^#^*p* < 0.050, ^##^*p* < 0.01 compared with the lowEBMIL group on octreotide days.

*PCM* Paracetamol, *bp* Beats per min, *RER* Respiratory exchange ratio.

Fasting and pre-meal C-peptide concentrations were higher in LowEBMIL, but AUCs after breakfast (Table 2) and ad libitum meals (Supplementary Table 1) were similar between groups on placebo days when adjusting for pre-meal concentrations. Octreotide lowered basal and postprandial C-peptide concentrations in both groups, but LowEBMIL had slightly higher C-peptide concentrations (peak and AUC with pre-meal correction) after breakfast on octreotide days (Table 2).

#### Glucagon (Fig. 2C)

Fasting and basal concentrations of glucagon did not differ significantly between groups. However, LowEBMIL had a higher glucagon response compared with HighEBMIL after breakfast (Table 2) and the ad libitum meal (Supplementary Table 1) on placebo days. Octreotide lowered glucagon in both groups reaching comparable concentrations in relation to breakfast and ad libitum lunch.

#### Intestinal nutrient entry (Fig. 2D)

Time to peak of paracetamol was similar between groups on placebo days and was similarly delayed by octreotide in both groups (Table 2).

#### Pulse rates (Fig. 2E) and blood pressure

Neither fasting nor basal pulse rates differed between groups. On placebo days, breakfast resulted in a similar 15–20 beats-per-minute (bpm) rise in both groups (Table 2), whereas peak pulse rate after the ad libitum meal tended to be higher in LowEBMIL (73 bpm [68;79] vs 66 [63;71], median [IQR], *p* = 0.079) (Supplementary Table 1). Octreotide lowered basal pulse rates and diminished postprandial peaks similarly in both groups. Blood pressure (BP) did not differ between groups, and octreotide increased basal, post-breakfast and post-lunch BP (systolic and diastolic) similarly in groups (data not shown).

#### Indirect calorimetry

RERs (basal and postprandial) did not differ between groups on placebo days and were lowered significantly by octreotide in both groups (Table 2).

## Discussion

We investigated the role of gut hormones for appetite control in women with primary WL failure after RYGB by comparing with carefully matched women with successful WL after surgery. We hypothesized that patients with WL failure would be characterized by lower postprandial secretions of the anorexigenic hormones GLP-1 and PYY (primary endpoint) based on studies demonstrating that GLP-1 and PYY actions inhibit food intake after RYGB [16] and observations of lower postprandial concentrations in suboptimal WL responders compared with good WL responders after RYGB [10, 14, 18]. In contrast to our hypothesis, patients with primary WL failure had similar GLP-1 and PYY concentrations after a fixed breakfast meal compared with patients with successful WL. Also, postprandial CCK concentrations did not differ between groups which is important since CCK receptor activation may be required for GLP-1 induced satiation, at least in rodents [26]. Fasting ghrelin concentration was lower in the primary WL failure group as also observed in previous WL response studies [14, 18, 19], consistent with the higher body weight [27], but the postprandial suppression of ghrelin did not differ between the groups contrasting with previous findings [10, 14, 18, 19]. Fasting leptin concentration was higher in the WL failure group consistent with higher fat mass, but contrasting with previous findings [20]. Ad libitum food intake did not differ between the groups on placebo days. Importantly, however, we found that inhibition of hormone secretions by octreotide did not affect ad libitum food intake in the patients with primary WL failure after RYGB, while octreotide increased ad libitum food intake by 23% in patients with successful WL. Increased ad libitum food intake and brain reward responses after octreotide administration have been reported after RYGB, but not after gastric banding [10, 17], and moreover the response to octreotide has been shown to be preserved in patients with weight regain after RYGB [14]. Thus, an absent effect of octreotide on ad libitum food intake may be a particular trait of patients with primary WL failure after RYGB.

Daily number of steps measured by blinded pedometers did not differ between the groups in line with some [28], but not all [29] studies using objective measures. Moreover, basal energy expenditure was higher in patients with primary WL failure consistent with larger lean mass in accordance with previous results [18] and as expected from measurements of 24-hour energy expenditure after RYGB [11]. Accordingly, differences in food intake seem to underlie the different WL in the two groups post-RYGB. This could, however, neither be detected on basis of the ad libitum food intake on the placebo day nor with 4-days food registration at home. Notably, both groups have undergone dietary consultations in relation to their surgery and may be equally aware of the optimal post-RYGB diet, as also reflected in the food registration, where energy intake and macronutrient composition including a high protein content were recorded similarly by the two groups. The postprandial increment in pulse rates as an indicator of early dumping did not differ between groups in accordance with previous findings [18]. Of notice, the postprandial pulse increment was abolished by octreotide in line with after administration of the somatostatin analogue pasireotide [30] likely explained by the suppression of hormones [30] or changed splanchnic blood flow after octreotide [31]. Interestingly, the patients with primary WL failure after RYGB reported lower satiety scores and less suppression of hunger scores after breakfast on placebo days despite similar postprandial concentrations of GLP-1, PYY, CCK and ghrelin compared with patients with successful WL. Postprandial glucagon concentrations were higher in the WL failure patients, possibly a consequence of body weight differences where a slightly higher HOMA2-IR could indicate higher liver fat content, which in turn may affect glucagon concentrations [32].

Hence, the parameters discriminating patients with primary WL failure from patients with successful WL after RYGB in this study were related to appetite control and the regulation by gut hormones as demonstrated by two findings: An absent effect on ad libitum food intake after inhibition of gut hormone secretions and an attenuated effect on postprandial satiety and hunger sensations despite similar postprandial gut hormone profiles. Thus, an impaired central sensitivity towards the anorexigenic effect of gut hormones might contribute to primary WL failure after RYGB. The underlying mechanisms explaining the extent of weight loss difference between the two groups are incompletely identified by this study, but it is of priority to clarify whether the impaired central anorectic response to gut hormones is a trait that potentially could be identified preoperatively thus sparing patients from surgery.

In this study, neither common variants in the genes encoding GLP-1, PYY and their receptors nor a polygenic BMI score discriminated patients with primary WL failure from patients with successful WL after RYGB, but in larger studies, whole genome sequencing should be considered for analysis of underlying genetic factors including rare genetic variants. Also worth investigating is the response to GLP-1R agonist (GLP-1RA) treatment. The GLP-1 RA liraglutide induces WL in patients with weight regain after RYGB [33, 34], but has not been investigated in patients with primary WL failure. In WL trials, 5–10% of patients are non-responders to high dose GLP-1RA treatment [35, 36] but since the preoperative WL response to a GLP-1RA does not seem to predict post-RYGB WL [37] this can hardly be used to select patients for surgery. Post-bariatric [38], but not pre-bariatric [39], behavioral interventions may add 2%-points of EBMIL [38] but whether this is also the case for patients with primary WL failure is unknown.

The strengths of this study are: the exclusive focus on patients with primary WL failure, and not weight regain, after RYGB, the specific prespecified primary and secondary hypotheses and the careful 1:1 matching between individuals, which was not done systematically in previous studies [10, 18, 19]. A limitation is the weight adjusted octreotide dose which might explain a slightly higher concentration of GLP-1 and a similar tendency for ghrelin after the ad libitum meal in HighEBMIL. Of note, AUCs were suppressed by octreotide compared with placebo and concentrations at initiation of the ad libitum meal were similar between groups. Nevertheless, higher GLP-1 concentrations in the HighEBMIL group would in theory imply that the (higher) ad libitum meal intake after octreotide was a conservative estimate; conversely, higher ghrelin concentrations might affect results oppositely.

In this study, primary WL failure after RYGB was not explained by impairments in the secretion of appetite regulating gut hormones or by variants in the genes for GLP-1, PYY or their receptors. However, inhibition of hormone secretions with octreotide increased food intake only in patients with successfully maintained WL after RYGB, whereas the effect was absent in patients with primary WL failure. Thus, an impaired central anorectic response to gut hormones could contribute to primary WL failure after RYGB.

### Supplementary information

Supplemental tables and figures

## Data Availability

The datasets from the study are available from the corresponding author on reasonable request.
