# Supplementary material for: Primary weight loss failure after Roux-en-Y gastric bypass is characterized by impaired gut-hormone mediated regulation of food intake
Source: Int J Obes (Lond). 2023 Aug 31;47(11):1143–51. doi: 10.1038/s41366-023-01372-8 (PMC10599997; doi:10.1038/s41366-023-01372-8)

**Supplementary table 1:**

**Appetite regulating hormones and measures of glucose metabolism in response to the ad libitum lunch meal**

|  | **Primary WL failure**  **(LowEBMIL)**  **Placebo** | **Successful WL**  **(HighEBMIL)**  **Placebo** | **P-value** | **Primary WL failure**  **(LowEBMIL)**  **Octreotide** | **Successful WL**  **(HighEBMIL)**  **Octreotide** | **Main Oct** | **Group x Oct** |
| --- | --- | --- | --- | --- | --- | --- | --- |
| GLP-1 at t=240 (pmol/L) | 7 [5;12] | 9 [5;11] | 0.749 | 4 [1;8] | 6 [4;7] | <0.001 | 0.351 |
| GLP-1 AUC_240-300_  (pmol·L^-1^·min) | 926  [716;1108] | 645  [446;1129] | 0.291  0.144^a^ | 244 **  [203;499] | 386** #  [266;435] | <0.001  <0.001^a^ | 0.010  0.009^a^ |
| GLP-1 peak (pmol/L) | 21 [15;26] | 17 [11;27] | 0.406 | 6** [4;10] | 9* [6;13] | <0.001 | 0.026 |
| PYY at t=240 (pg/mL) | 136 [102;155] | 136 [119;170] | 0.127 | 70 [50;90] | 80 [61;97] | <0.001 | 0.712 |
| PYY AUC_240-300_  (pg·mL^-1^·min) | 8850 [7160;10441] | 9296 [7946;10819] | 0.573  0.619^a^ | 4452  [3062;5849] | 4829 [3903;6233] | <0.001  <0.001^a^ | 0.719  0.306^a^ |
| PYY peak (pg/mL) | 159 [140;196] | 166 [140;206] | 0.795 | 94 [54;115] | 87 [64;121] | <0.001 | 0.735 |
| CCK at t=240 (pmol/L) | 0.6 [0.4;1.3] | 0.5 [0.3; 0.6] | 0.363 | 0.1 [0.1;0.3] | 0.2 [0.1;0.3] | <0.001 | 0.316 |
| CCK AUC_240-300_  (pmol·L^-1^·min) | 121  [70;146] | 46  [32;101] | 0.016  0.089^a^ | 13**  [8.8;20] | 15**  [6.0;25] | <0.001  <0.001^a^ | 0.041  0.150^a^ |
| CKK peak (pmol/L) | 3.0 [1.7;4.1] | 1.0 [0.8;2.7] | 0.017 | 0.3 [0.3;0.5] | 0.4 [0.1;0.6] | <0.001 | 0.076 |
| Ghrelin at t=240 (pg/mL) | 40 [32;63] | 40 [24;48] | 0.824 | 26 [17;30] | 30 [18;37] | <0.001 | 0.352 |
| Ghrelin AUC_240-300_  (pg ·mL^-1^·min) | 1973  [1521;3135] | 2055  [1095;3158] | 0.827  0.422^a^ | 1181  [818;1425] | 1620 [1393;2269] | <0.001  <0.001^a^ | 0.079  0.114^a^ |
| Ghrelin nadir (pg/mL) | 22 [16;30] | 23 [12;40] | 0.855 | 10** [4;16] | 19# [12;30] | <0.001 | 0.027 |
| VAS_satiety_ at t=240 (mm) | 25 [18;49] | 49 [23;70] | 0.021 | 35 [23;52] | 51 [27;64] | 0.308 | 0.150 |
| VAS_satiety_ AUC_240-300_ (mm) | 4238 [3728;4451] | 4380  [3818;5160] | 0.034  0.307^a^ | 4050  [3608;4537] | 4485 [3965;5299] | 0.862  0.810^a^ | 0.989  0.447^a^ |
| VAS_hunger_ at t=240 (mm) | 62 [27;79] | 45 [17;66] | 0.151 | 51 [36;70] | 46 [32;60] | 0.604 | 0.132 |
| VAS_hunger_ AUC_240-300_  (mm ·min) | 1298  [679;1489] | 840  [368;1245] | 0.079  0.848^a^ | 1110  [671;1493] | 1058 [619;1605] | 0.199  0.148^a^ | 0.228  0.586^a^ |
| Glucose at t=240 (mmol/L) | 4.6 [4.4;4.9] | 4.6 [4.4;4.8] | 0.991 | 7.3 [6.6;8.3] | 7.6 [6.0;8.8] | <0.001 | 0.831 |
| Glucose AUC_240-300_  (mmol·L^-1^·min) | 358  [338;395] | 356  [316;378] | 0.625  0.482^a^ | 440**  [400;480] | 504**  [411;549] | <0.001  <0.001^a^ | 0.163  0.038^a^ |
| Glucose peak (mmol/L) | 6.6 [6.1;7.5] | 6.4 [5.6;7.1] | 0.494 | 7.6 [7.0;8.4] | 8.6 [7.3;9.5] | <0.001 | 0.062 |
| C-peptide at t=240 (pmol/L) | 692 [585;1044] | 474 [399;538] | <0.001 | 866 [558;1045] | 561 [375;663] | 0.199 | 0.735 |
| C-peptide AUC_240-300_  (nmol·L^-1^·min) | 90 [71;102] | 40 [37;72] | 0.002  0.318^a^ | 48 [40;76] | 42 [32;50] | <0.001  <0.001^a^ | 0.295  0.354^a^ |
| C-peptide peak (pmol/L) | 1766 [1410;2022] | 808 [707;1485] | 0.003 | 811 (712;1385] | 760 [594;903] | <0.001 | 0.171 |
| Glucagon at t=240 (pmol/L) | 9.0 [3.8;12] | 4.0 [1.0;8.5] | 0.012 | 1.0 [1.0;1.0] | 1.0 [1.0;1.0] | <0.001 | 0.168 |
| Glucagon AUC_240-300_  (pmol·L^-1^·min) | 510  [173;709] | 326  [105;441] | 0.038 0.444^a^ | 60  [60;90] | 60  [60;60] | <0.001  <0.001^a^ | 0.667  0.442^a^ |
| Glucagon peak (pmol/L) | 11 [4.0;13] | 6.5 [2.0;9.3] | 0.062 | 1.0 [1.0;2.0] | 1.0 [1.0;1.0] | <0.001 | 0.835 |
| Pulse rate peak (bpm) | 73 [68;79] | 66 [63;71] | 0.079 | 63 [57;70] | 60 [56;67] | <0.001 | 0.229 |

Median [IQR]. ^a^Model adjusted for pre-meal concentrations/levels, *p<0.050, **p<0.010 compared with placebo day, #p<0.050, ## p<0.01 compared with the lowEBL group on octreotide days.

**Supplementary table 2:**

**Functional annotation of variants in the GCG, GLP1R, PYY and NPY2R genes.**

| **Gene variant type** | **GCG** | **GLP1R** | **PYY** | **NPY2R** |
| --- | --- | --- | --- | --- |
| 3_prime_UTR_variant | 2 | 40 | 45 | 23 |
| 5_prime_UTR_variant | 1 | 6 | 16 | 2 |
| downstream_gene_variant | 23 | 69 | 134 | 13 |
| intergenic_variant | 76 | 500 | 92 | 781 |
| intron_variant | 514 | 1442 | 573 | 740 |
| missense_variant | 3 | 16 | 13 | 3 |
| non_coding_transcript_exon_variant | 8 | 21 | 69 | 44 |
| splice_acceptor_variant | 1 | 0 | 1 | 0 |
| splice_donor_variant | 1 | 0 | 2 | 1 |
| splice_region_variant | 0 | 1 | 6 | 4 |
| synonymous_variant | 3 | 15 | 14 | 5 |
| upstream_gene_variant | 17 | 89 | 181 | 30 |
| **Total number of variants for analysis** | **649** | **2199** | **1146** | **1646** |

**Supplementary table 3:**

**Variants in the extended gene regions for GCG, GLP1R, PYY and NPY2R (500kb upstream from the transcriptional startsite, to 250kb downstream from the transcriptional end) when testing the effect of being a carrier on the probability of being in the HighEBMIL versus the LowEBMIL group.**

| Gene | GCG | GLP1R | PYY | NPY2R |
| --- | --- | --- | --- | --- |
| Display range: | chr2:162749391-163508757 | chr6:38516556-39309079 | chr17:41780100-42581813 | chr4:155594874-156388228 |
| Reference SNP: | chr2:162913811 | chr6:39125675 | chr17:42332803 | chr4:156018683 |
| Min P−value: | 4.72E−2  [chr2:162913811] | 5.68E−2  [chr6:39125675] | 6.37E-2  [chr17:42332803] | 3.35E−2  [chr4:156018683] |
| Max P−value: | 9.99E−1  [chr2:162959756] | 9.99E−1  [chr6:38897030] | 10E-1  [chr17:42401708] | 9.99E−1  [chr4:155647114] |

**Supplementary figure 1:**

**Regional plots of the p-values for each of the variants in the extended gene regions for GCG (A), GLP1R (B), PYY (C) and NPY2R (D) (500kb upstream from the transcriptional startsite, to 250kb downstream from the transcriptional end) when testing the effect of being a carrier on the probability of being in the HighEBMIL versus the LowEBMIL group.**

**Supplementary Figure 1A: GCG**


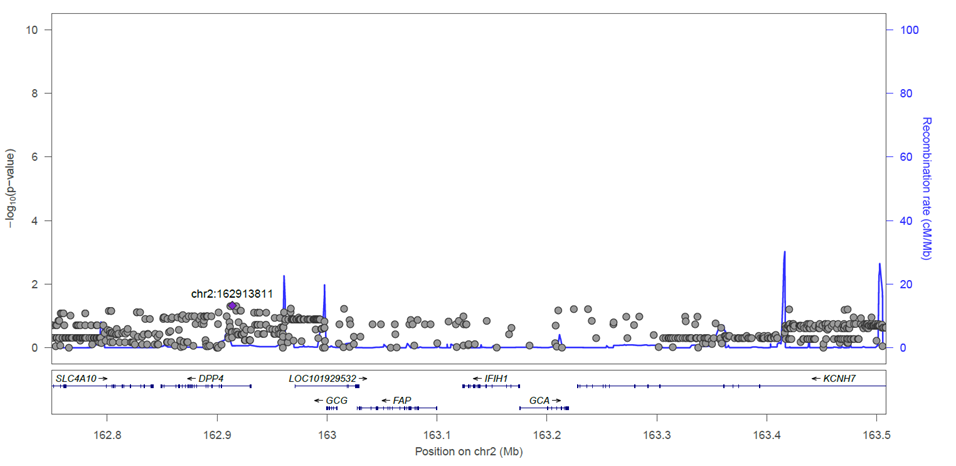


**Supplementary Figure 1B: GLP1R**


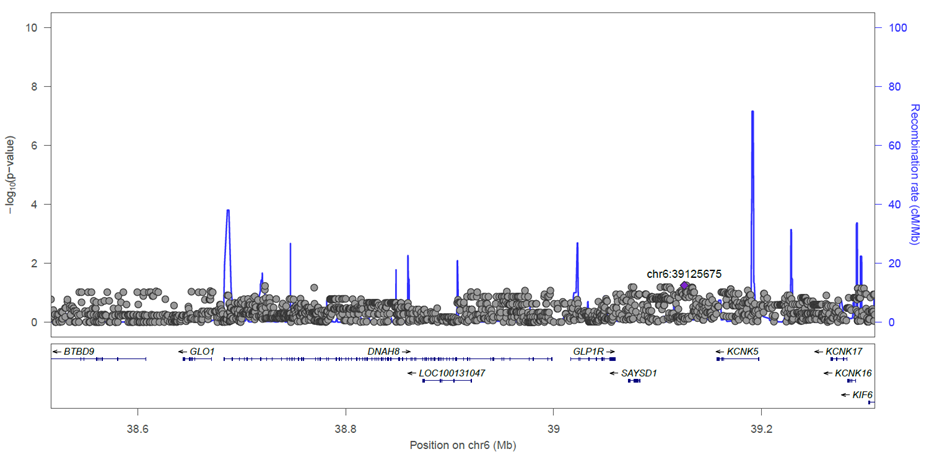


**Supplementary Figure 1C: PYY**


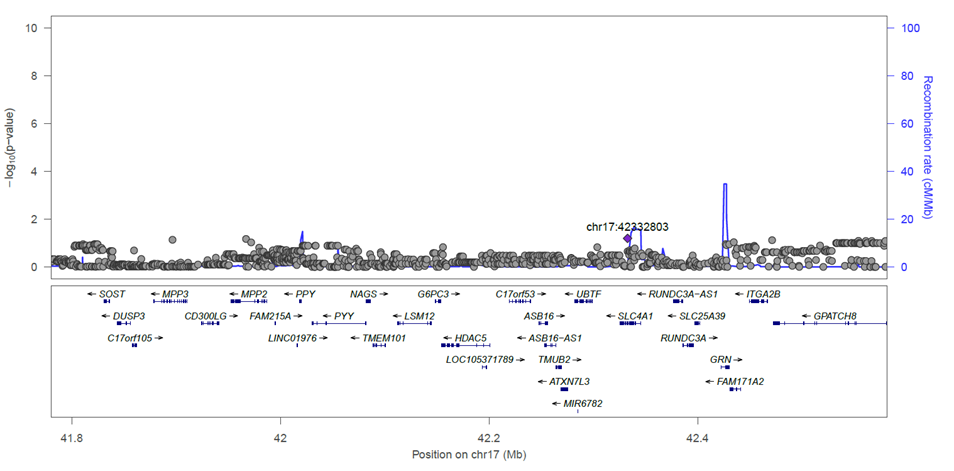


**Supplementary Figure 1D: NPY2R**


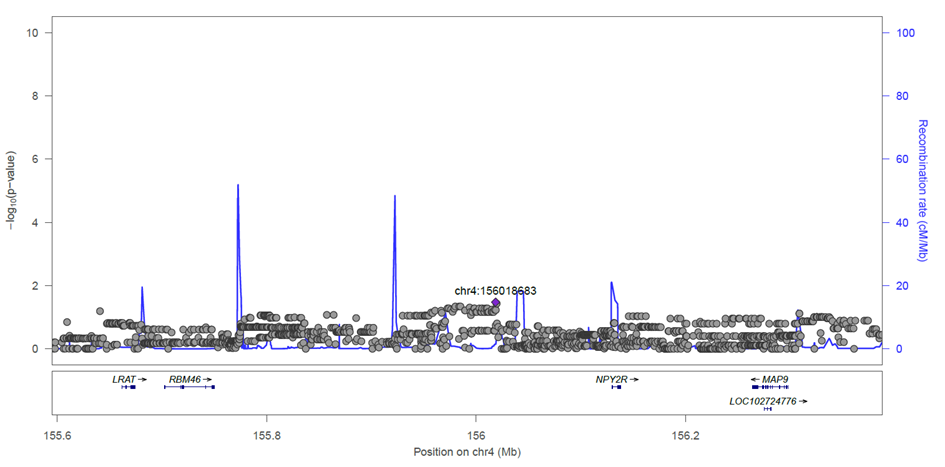

Supplement: Supplementary file 1 — Supplemental tables and figures [file 41366_2023_1372_MOESM1_ESM.docx]
